# Dietary Costs among Midwestern Adult Food-Pantry Users by Food-Security Status

**DOI:** 10.3390/nu15030680

**Published:** 2023-01-29

**Authors:** Agustina Fainguersch, Aaron J. Dewar, Lacey A. McCormack, Heather A. Eicher-Miller

**Affiliations:** 1 Department of Nutrition Science, Purdue University, West Lafayette, IN 47907, USA; 2 Department of Statistics, Purdue University, West Lafayette, IN 47906, USA; 3 Avera Research Institute, Sioux Falls, SD 57108, USA

**Keywords:** food security, dietary quality, dietary expenses, food insecurity, secondary analysis

## Abstract

Evidence of the relationship between dietary cost, diet quality, and socio-economic status is mixed. No studies have directly evaluated food-security status and dietary cost. This study investigated whether food-pantry clients with low and very low food-security status had less expensive daily diets than food-secure clients by comparing total cost, cost per gram, and cost per calorie of total daily dietary intake both per person and by individual food item, followed by evaluations of each food group. Mixed-model regression and Tukey–Kramer comparisons were used to compare food-security groups. There was no clear association between food-security status and cost of daily diet. Analyzed per person, total price and price per gram showed significant differences between low food-secure and food-secure groups. When analyzing individual food items, prices per calorie were significantly different between food-secure and very low food-secure groups. The directionality of the relationships by food-security status was inconsistent. Per person, those with lower food security had lower mean prices, and for individual foods this association was reversed. Therefore, the metric of food cost and the unit of analysis are critical to determining the relationship between food-security status and dietary cost.

## 1. Introduction

Food insecurity is a situation households experience when there are not enough money or resources to maintain the quality or quantity of foods needed for all members to have an active, healthy life [1]. In 2021, approximately 10 percent of all US households experienced food insecurity, encompassing both low and very low food-secure groups. About 4 percent of US households experienced very low food security with insufficient dietary intake and instances of disrupted eating patterns caused by financial burdens and lack of resources [1]. In contrast, the 6 percent of households experiencing low food security reported few, if any, reductions in dietary intake but still experienced reductions in dietary quality and barriers to obtaining sufficient food [1]. Poor dietary quality is a hypothesized link bridging food insecurity and negative health outcomes. Studies have shown that food insecurity is associated with an increased risk of heart disease as well as hypertension, diabetes, and obesity in middle-aged adults [2,3,4,5]. 

Lack of resources to purchase a diet of sufficient quality and the expense of “healthful” food have been used as explanations of the relationship between food insecurity and poor dietary quality among low-income groups [6,7,8]. “Healthful” foods can be defined as those that fulfill the 2020–2025 Dietary Guidelines for Americans (DGA), which state that adults should consume a variety of nutrient-dense foods in vegetable, fruit, grain, dairy, and protein groups, while staying within calorie ranges, and limit consumption of added sugars, saturated fats, sodium, and alcohol [9]. Some studies evaluating dietary costs reveal that higher-quality diets are more costly than lower-quality diets, suggesting that low-income groups purchase and consume more unhealthy foods that are lower in price [10,11,12,13]. In addition, results showing that energy-dense foods high in refined grains and added sugars and fats are more accessible and less expensive than less energy-dense foods, such as fresh fruits and vegetables, have been reported [10]. However, other studies have suggested the opposite, reporting that healthful foods cost the same or even less than less healthful foods [14,15,16]. These mixed results have included low-income groups, but having a low income is not specific to the situation of food insecurity since the two metrics are not always directly correlated [17]. Food security characterizes being able to maintain food access; therefore, comparing expenses while accounting for dietary quality among those who are able and not able to maintain food security is salient to determining whether costs are responsible for obvious differences between food-secure and food-insecure situations. Investigation of this relationship among food-pantry participants is ideal because food-pantry users represent a mixed group: some may be food-insecure and reach out to the pantry to obtain additional foods, while others may be food-secure but could be utilizing pantries as a tool to improve their food supply and the health of their households [2]. 

Through this analysis, we seek to analyze the problem of how food-security status could be linked to dietary cost. Food-insecure and food-secure food-pantry clients are expected to have daily diet costs that do not differ in terms of total cost, cost of diet per calorie, and cost of diet per gram for total daily dietary intake and for food-group intake, when analyzed both at the individual level and at the food-item level. The results of this investigation may be used to inform interventions, such as the Supplemental Nutrition Assistance Program (SNAP), the Special Supplemental Program for Women, Infants and Children (WIC), and the National School Lunch Program, to improve dietary intake among a vulnerable sub-population of food-pantry users. Therefore, the objectives of this study are to: (1) determine the daily dietary costs of adult food-pantry clients (both per person and per food) in the Midwest according to food-secure, low food-secure, and very low food-secure status for total diet and by food group and (2) evaluate whether differences in dietary costs for total diet and by food group exist between clients of various food-security status (per person) when dietary quality is held constant and among the foods reported by clients of these various food-security status groups (per food item). Cost in this study will not be the true cost paid by the individual (or not paid in the case of food-pantry foods) but rather the value of the foods as determined by the US Department of Agriculture. 

## 2. Materials and Methods 

### 2.1. Study Design 

This secondary analysis utilizes the 2014 baseline dietary intake data from food-pantry client participants in the Voices for Food project (VFF), a collaborative intervention delivered through universities in six Midwestern states: Indiana, Michigan, Missouri, Nebraska, Ohio, and South Dakota. The long-term goal of VFF was to increase Midwestern food-pantry users’ food security and diet quality, a complete description of which has previously been published [18]. A total of 24 rural pantries (four from each state) were included in the study, all recruited based on having elevated county poverty rates of 16% or higher in 2011, as well as having a non-metro county classification [18,19]. A total of 613 participants were recruited and provided information regarding their demographics, living situations, economic status, diet, food-accessibility, and other factors. Each participant completed an in-person baseline 24-h dietary recall dietary assessment with a trained interviewer using the Automated Self-Administered 24-Hour Dietary Assessment (ASA24™-2014), a web-based, manual-entry dietary recall system [20]. Some participants completed up to two more self-completed recalls on non-consecutive days, at least one of which was at a weekend. However, only the first weekday recall was used in this analysis since not all participants completed multiple days of recalls. Newer versions of food-pantry client data of equivalent caliber are not available.

### 2.2. Participant Recruitment

Participants were recruited from August to November in 2014, either by trained research staff or through flyer promotion of the study at each selected pantry that met the previously described conditions [18,19]. Research staff performed screening for eligibility at food pantries during food distribution, ensuring that the potential participants were English-speaking, aged 18 or older if they were from five of the six states or 19 or older if they were from Nebraska, and that they had used the food pantry at least one other time in the past year. All participants provided verbal or written consent, and the universities’ Institutional Review Boards provided approval for the study. With exclusions of incomplete and subsequent recalls, the final analysis of the initial 24-h recall included 588 participants. 

### 2.3. Assessment and Data Sources

#### 2.3.1. Food-Security Status and Other Characteristics

Demographic information as well as characteristics of pantry use for each participant were obtained through an interview with a research staff member at the participating pantries, and a 54-item questionnaire was delivered either electronically or via paper copy. The questionnaire inquired about participant characteristics and use of food pantries, the following of which were utilized in the analysis: state of residency, sex, age, ethnicity, race, highest level of education, work status, household income, and pantry-client participation in the following food-assistance programs: SNAP, Meals on Wheels, Soup Kitchens, and WIC. The food-security status of participants was queried via the US Household Food Security Survey Module, an 18-question survey that asks about the conditions, experiences, and behaviors related to household access to food due to limited resources. This was included in the participant questionnaire and was used to categorize responses into four levels: very low food-secure, low food-secure, marginally food-secure, and food-secure [21,22]. For the purposes of this study, marginally food-secure and food-secure were grouped into a single “food-secure” category. All participants received a $10 grocery gift card as compensation for successful completion of the interview and questionnaire. 

#### 2.3.2. Participant Dietary Intake Data, Dietary Quality, and Food Prices 

In the ASA-24 dietary recall, each food reported was assigned a unique 8-digit USDA food code. This food code was matched with the same USDA food code in the 5th version of the Food and Nutrient Database for Dietary Studies (FNDDS) [23], chosen to align with the years of data collection, to obtain the energy and food-group classifications of the foods consumed by each participant. FNDDS supplies information on energy and food composition, which was used for the diet price calculations in this secondary analysis. 

The USDA Center for Nutrition Policy and Promotion (CNPP) Food Price Database from 2003–2004 [24], the most recent version available, was used for the price calculations of the foods consumed by each participant. The first version of the CNPP price database was published in 2001–2002 and attached price data to the National Health and Nutrition Examination Survey (NHANES) dietary recall data. Foods reported in NHANES (in their as-consumed form) were first broken down into their ingredients using FNDDS recipes. Then, the ingredients were transformed into number of as-consumed grams using the National Nutrient Database for Standard Reference (NNDSR), which contains information regarding moisture loss and gain in cooking. The costs of these ingredients were then calculated by averaging the prices paid by consumers who participated in the Nielsen Homescan™ Consumer Panel [25]. The CNPP price database lists the price per 100 g of foods by USDA food code. In order to link price information to the dietary data reported by VFF participants in this study, the USDA food codes within the CNPP price database and FNDDS were matched for each food and beverage consumed, with the exception of 85 food items for which the food codes did not match due to incompatible versions of the FNDDS and CNPP price databases. For those 85 food items, recoding was performed, whereby a similar food code in the CNPP price databased was assigned to each of the foods. Then, the quantities of each food reported in the 24-h dietary recall were converted into units of 100 g. Next, the total daily costs of food consumed, costs per gram, and costs per calorie were determined in two ways: by food as the unit of analysis and by person as the unit of analysis. For food as the unit of analysis, individual foods were categorized by the food-security status of the individual who consumed the food, and the cost totals for all foods consumed by a particular food-security status group were calculated. The cost means and standard deviations per food item out of all items consumed by that group were estimated. With persons as the units of analysis, daily totals, means, and standard deviations of costs per person within each food-security status group were calculated, and these calculations were per all of the foods (or foods in a food group) reported by that individual. 

**Figure 1:**
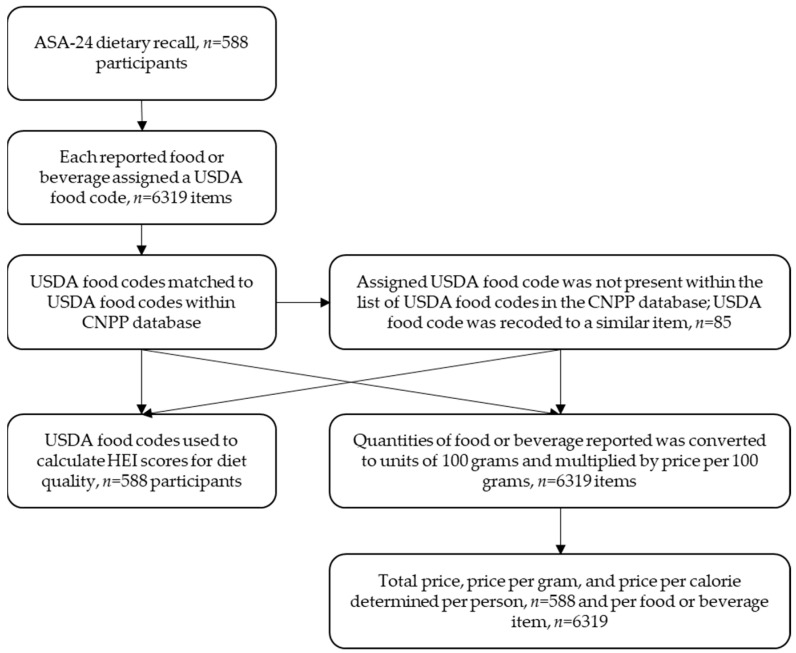
Flowchart describing the process used to convert ASA-24 dietary recall data to total price, price per gram, and price per kilocalorie per person and per food or beverage utilizing USDA food codes and the CNPP price database.

The ASA24-2014 system is linked to the Food Patterns Equivalents Database 2011–2012 to allow the calculation of Healthy Eating Index (HEI)-2010 total and component scores. The HEI-2010, a scoring metric created by the USDA to determine the adherence of diets to the dietary guidance of the DGA, was used to derive dietary quality measures for food-pantry clients. The HEI-2010 includes nine “adequacy” categories (total fruit, whole fruit, total vegetables, greens and beans, whole grains, dairy, total protein foods, seafood and plant proteins, and fatty acids), where a higher score indicates higher consumption of a particular type of food. There are also three “moderation” categories (refined grains, sodium, and empty calories) which are reverse-scored so that a higher score indicates lower consumption. The scores from all twelve categories are combined to produce a total HEI score classifying an individual’s overall dietary quality, with higher scores indicating a mix of foods that aligns more closely with the guidance of the DGA, 100 being the highest possible score [26]. The analytic process is outlined in Figure 1.

### 2.4. Statistical Analysis

Participant characteristics among food-security groups were compared using chi-square analyses and included state of residency, age, sex, race, ethnicity, household income, employment status, diet quality (using HEI-2010 scores), and meal-assistance program participation. Significantly different characteristics among food-security groups, which included state, age, income, HEI-2010 score, and meal-assistance program use, were controlled for in all of the regression models. Sex and ethnicity were also controlled for in the models, although they were not significant because they were identified as appropriate adjustments. The independent variable of interest was food-security status classified into three groups: very low food-secure, low food-secure, and food-secure. The dependent variables representing the costs of foods consumed were total price, price per calorie, and price per gram. These three cost metrics were analyzed per person and by individual foods both for overall diet and for the following food groups: dairy, fruits, grains, protein, and vegetables. These five food groups represent the food groups recommended for inclusion by the 2020–2025 DGA [9]. Residual plots of dependent variables were evaluated for normality, and the dependent variables were log-transformed to meet model assumptions and smooth out variances. Therefore, the *p*-values displayed are for log-transformed data, while means and standard deviations are shown for the untransformed data to allow for realistic applications. A linear mixed-effects model was used to compare the cost or price of foods consumed by food-security group, using separate models for the various price variables (total price, price per gram, and price per kilocalorie, and by person or by food as the unit of analysis). Participant ID and region were included as random effects in the per-person analysis, and region was used as a random effect in the individual food analysis. Statistically discernible differences were assumed with *p*-values < 0.05. For dependent variables, where significant differences were observed, Tukey–Kramer pairwise comparisons were performed on the log-transformed data in order to determine which two groups differed, once again using an alpha = 0.05 to indicate statistical significance. SAS 9.4 was used to perform all statistical analyses. 

## 3. Results

### 3.1. Participant Characterisitics

The pantry clients were predominately white (78%), female (60%), aged 45–64 (37%), and classified as food-insecure (77%), with 30% experiencing low food security and 47% experiencing very low food security (Table 1). When stratified by food-security status, there were significant differences observed for state, age, income, and meal-assistance program use. A greater proportion of food-pantry clients above 65 years old were food-secure (31%) compared with other food-security groups. A greater proportion of food-insecure pantry clients reported using meal-assistance programs when compared with food-secure clients (77%). Of those who used SNAP, WIC, Meals on Wheels, and soup kitchens, larger percentages were food-insecure (82%, 77%, 84%, and 85%, respectively). There was also a significant difference in dietary quality between food-security groups, with food-secure participants having the highest HEI score at 45 and very low food-secure participants having the lowest score at 42 on a 100-point scale.

### 3.2. Mean Total Price Metric Differences by Food-Security Group

Analyzing the three price metrics (total price, price per gram, and price per calorie) for the 24-h recall period by mean cost of daily diet per person and mean cost of diet for individual foods showed varying significant differences between food-secure, low food-secure, and very low food-secure groups (Table 2). Total price and price per gram values among the log-transformed data per person were statistically different between groups (*p* = 0.004 and *p* = 0.03, respectively). For both categories, Tukey pairwise comparison showed a difference between low food-secure and very low food-secure groups. For total price per person, the low food-secure group had a higher untransformed mean ($2.89) than the very low food-secure group ($2.27). Price per gram per person followed the same trend, with low food-secure participants averaging $0.0015 per gram and very low food-secure participants averaging $0.0012 per gram. Regarding the estimates by individual food, prices per calorie were statistically different between groups (*p* = 0.006), but this time the difference was between the very low and food-secure groups, the food-secure group having a lower untransformed mean price per kilocalorie ($0.0038) compared to the very low food-secure group ($0.0043). 

### 3.3. Mean Food Group Total Price Metric Differences by Food-Security Group 

Per person, the results showed that total price of fruit was notably different among food-security groups (*p =* 0.04) (Table 3). Differences were apparent between the low food-secure ($0.47) and food-secure groups ($0.38), with the latter having a lower untransformed mean. Prices per gram of fruit per person (Table 4) were also different between food-security groups (*p* = 0.01), with the very low food-secure group showing a higher untransformed mean at $0.0021 per gram compared to $0.0018 for the low food-secure group. A similar result was noted for price per gram of fruit for individual foods (*p* = 0.03), also between very low and low food-secure groups (Table 4), with very low food-secure participants having a higher untransformed mean ($0.022) compared to low food-secure participants ($0.0019). In other results of the analysis of individual foods, prices per gram of vegetables were different (*p* = 0.0001) (Table 4). This time, low food-secure and food-secure groups were different. Food-secure participants had a lower individual food price per gram mean for vegetables ($0.0025) than the low food-secure ($0.0029) group. No evidence of significant differences was observed between any food-security groups regarding prices per calorie (Table 5). 

## 4. Discussion

This study evaluated costs, adjusting for dietary quality, among food-pantry clients by food-security status. The results showed that the relationship between dietary cost and food security was mixed, and that higher dietary costs were not consistently linked to food security or level of food insecurity. Although there were significant differences between certain dietary cost scenarios, the inconsistency among the different price metrics and units of analysis showed that there was no clear association between food-security status and cost of diet among adult food-pantry users when dietary quality was held constant. When considering all foods together, total price and price per gram were significantly different per person, and price per calorie was different for individual foods, but the two food-security groups responsible for the significance were not consistent. In the per person analysis of total price and price per gram, very low and low food-secure groups showed evidence of being significantly different, with the latter having a higher mean. However, individual food analysis showed a difference between very low and food-secure groups, with the latter having the lower mean. By performing the same statistical tests on the same data set per person and by individual food item and obtaining these varying results, the mixed relationship between these two variables was further exemplified. 

Analysis of individual food groups showed few significant differences between dietary cost metrics among food-secure groups, both per person and based on individual foods. The food groups found to exhibit differences were fruits and vegetables, which are commonly considered “healthy” food groups and foods that most Americans need to consume more of, as stated in the DGA [9]. Fruits and vegetables are unique because of their high water densities, which translate to lower calorie contents per gram. Additionally, fruit and vegetable food groups are characterized by episodic consumption, defined as a percentage of non-consumption greater than 5% among this sample of participants in the VFF study [27], which, when combined with high water densities, could be responsible for some of the underlying differences between these groups that were found through the analysis. Prior researchers have also concluded that the metric used to quantify the cost of diet can affect the conclusion as to whether healthier foods are more or less expensive than less healthy foods [28]. Lower-calorie foods were found to be more expensive when price was evaluated per calorie, which means that fruits and vegetables would be more expensive [28]. Accordingly, “moderation” foods high in calories, saturated fat, and added sugars tend to have a lower price per calorie. However, vegetables and fruits were found to be less expensive than “moderation foods” on the basis of edible weight [28]. Although this analysis found no significant differences between prices per calorie for any food group when comparing groups of differing food-security status, prices per gram of fruits and vegetables showed notable differences between food groups. In all cases of significant differences, the group with the lower food-security status of the two groups compared had a higher average cost per gram. This finding that very low food-secure participants had higher costs per gram for fruits and vegetables compared to low food-secure participants shows an increased need to investigate this relationship further, as prior research has focused on the relationship between lower socioeconomic status and intake of energy-dense foods high in refined grains, added sugars, and added fats in terms of calorie per gram and price per calorie [10], and the higher costs for “healthful” food groups seem to contradict this finding. If individuals in food pantries are selecting fruit and vegetable groups that should improve their dietary quality, then further investigation should focus on other nutritional factors that are contributing to adverse health outcomes.

Prior studies have investigated the link between income and dietary cost and quality. Some have shown that more expensive and higher quality diets were consumed by higher socioeconomic status groups with better health outcomes compared with individuals of lower socioeconomic status [13]. However, these studies focused on price per calorie analyses of nutrient-dense foods, particularly fruits and vegetables. Along with prior research [29], the analysis presented here examining prices per calorie also showed these less calorie-dense food groups to be more expensive and therefore potentially harder to obtain for individuals with lower food security. However, the results of the present study also showed that fruits and vegetables were two of the least expensive groups when total prices and prices per gram were evaluated. Therefore, the costs of foods within a food group depend on the way prices are evaluated. One possible consideration is that the actual form of the food (canned, fresh, frozen, etc.) is more significant with respect to cost than the food itself. In food-pantry settings, non-perishable items, such as canned goods, are often provided. Canned fruits and vegetables are affordable dietary options compared to their fresh counterparts for those of both food-insecure and food-secure status. For example, the CNPP price for cooked fresh spinach is $0.61 per 100 g compared to $0.32 for cooked frozen spinach and $0.28 for cooked canned spinach [24]. Similarly, raw apples are $0.22 per 100 g, whereas unsweetened applesauce is $0.15 per 100 g [24]. Canned and frozen fruits and vegetables may have other qualities, such as longer shelf-life, compared with fresh products. Alternatives such as these shelf-stable versions could be a cost-saving way to incorporate the recommended daily servings [9] of both fruit and vegetable food groups and improve dietary quality.

These results are relevant to modeling the minimum expenses for foods required to obtain a nutritionally adequate diet. The Thrifty Food Plan, for example, is a USDA-created food plan that estimates the cost of a healthy diet at a low price point, and it is used to determine SNAP benefit amounts for American households [30]. The Thrifty Food Plan represents an at-home diet for a family of four, accounting for the inflation of food prices through June of 2021, and defines $835.57 per month as a reference point for a sufficiently nutritious diet, which translates to $208.89 per person. Per day, this would be $6.73 for a 31-day month. This cost is approximately 2.5 times higher than any of the averages found in this analysis, even among food-secure participants, which may explain the low HEI scores and low dietary quality found in this sample of food-pantry clients, as the cost of their diet does not appear to be enough to support the recommendations of the DGA outlined in the Thrifty Food Plan. The mean total price of $2.27 for food-pantry clients with very low food security compared to $2.89 for those with low food security shows only a $0.52 difference per day. However, this translates to $3.64 per week, approximately $15.60 per month and $189.80 per year. This difference could be large enough to qualify one group for SNAP benefits but not another if the CNPP prices were adjusted for current-day inflation, as the difference is almost as large as the $208.89 per-month reference point modeled by the Thrifty Food Plan. With approximately 55% of food-insecure households participating in one or more of the federal assistance programs (SNAP, WIC, and the National School Lunch Program) [1], the difference between the low food-secure and very low food-secure groups should be noted and further investigated. However, this difference was only present between the food-insecure groups in one of the dietary cost scenarios and showed no significant difference in costs with food-secure clients in the per person analysis. Food-secure clients did have significantly different dietary costs, which were higher than the very low food-secure groups in the individual food analysis for price per kilocalorie at $0.0038 per kilocalorie and $0.0043 per kilocalorie. Although these differences seem minimal, the figure of $0.0005 for a 2000-calorie diet translates to $1.00 more per day and approximately $365 more per year for food-secure clients. 

### 4.1. Strengths 

By analyzing dietary cost in terms of total price, price per calorie, and price per gram, this secondary analysis was able to comparatively show various price metrics used in the prior literature regarding cost of diet and diet quality, all with a similar sample [10,11,12,13,14,15]. Additionally, the analysis added novelty by comparing these price metrics among groups according to food-security status rather than socioeconomic status, as not everyone of low socioeconomic status is food-insecure and vice versa. The analysis and conclusions are drawn from the results for a relatively large sample size (*n* = 588) from food pantries from a variety of Midwestern states, providing evidence that may be applicable to a larger population [29]. 

### 4.2. Limitations

Twenty-four-hour recalls are subject to errors by individuals, particularly under-reporting [31]. Furthermore, a single day’s recall does not represent an individual’s usual daily diet. However, the ASA-24 has been recognized as a tool that can capture approximately 80% of all foods and drinks actually consumed. The self-administered test is also subject to error, as some subjects choose items closer to the top of the search result list, limiting accuracy. Additionally, some respondents may not be able to locate the exact item they consumed, which could also result in misreporting [32]. The results of this analysis represent single days of dietary intake, and the mean prices estimated may not be assumed to represent the usual costs of diets yet can be used to estimate the costs of diets at a group level. The years included in the FNDDS and the CNPP price databases were not the same, as the CNPP was not updated for the year matched to the study data. As a result of the difference in years, not all foods in the clients’ dietary intake records had prices in the CNPP price database. Although recoding assigned a price, the food match may not have been perfect. Food prices tend to rise over time, meaning the actual means of the price metrics could have been higher than those reported in this study. Additionally, the study did not account for state-specific tax policies which could lead to variations in actual prices. The costs assigned here were likely not the actual costs for participants, as the true prices could have been influenced by region, store, coupons, and whether the food came from the food pantry or not. There were also large standard deviations for the untransformed means due to great variations amongst participants’ individual diets. Although the *p*-values were found after normalizing the data with a log transformation, the raw means could be impacted by these outliers, even with the large sample size. 

### 4.3. Conclusions

The results of this study did not establish a consistent relationship between the food-security statuses of food-pantry users and the costs of their diets. Evaluation of total prices, prices per calorie, and prices per gram, both by individual person and individual food item, yielded some significant relationships regarding food-security status, but the directionality varied according to the metrics and units of analysis used. Therefore, the metric of food cost and the unit of analysis are critical to determining the relationship between food-security status and dietary cost.

### 4.4. Recommendations and Future Directions

By analyzing the costs of diets using a variety of metrics and revealing an inconsistent relationship between price and food-security status, this study showed that the cost of diet does not have a direct relationship to food insecurity. Furthermore, dietary costs can not only vary according to the way that prices are calculated (as totals, by edible grams, or by kilocalories), but also by the unit of analysis used, by foods or by individuals. Further studies should focus on the interesting relationship between food-security status and the cost of fruits and vegetables in the diet, for example, by potentially investigating the frequency of fresh, canned, and/or frozen fruit and vegetable consumption among certain groups and its relationship to dietary quality. Although there was no direct relationship found between cost and food-security status, total costs per day for all three food-security groups in this study were lower than those modeled by the Thrifty Food Plan. Such findings could indicate a need for greater government funding and resources for low-resource groups in poorer areas of the country. Since the sample data were obtained from Midwestern states, it would also be beneficial to compare these data with those of pantries in more urbanized parts of the nation and see how dietary costs and quality compare.

## Figures and Tables

**Table 1:** Characteristics of a multistate sample of rural, Midwestern, adult emergency food-pantry clients by food-security status (*n* = 588).

	All Pantry Clients		Food-Secure		Low Food-Secure		Very Low Food-Secure		χ^2^
	*n*	%	*n*	%	*n*	%	*n*	%	*p*-Value
State									0.01 *
Total	588	100	133	23	177	30	278	47	
IN	157	27	30	23	42	24	85	31	
MI	99	17	17	13	34	19	48	17	
MO	141	24	35	26	37	21	69	25	
NE	50	9	10	7	24	13	16	6	
OH	79	13	20	15	19	11	40	14	
SD	62	10	21	16	21	12	20	7	
Age									0.0001 *
Total	502	85	115	23	157	27	230	39	
18–44 y	185	31	36	31	43	37	36	31	
45–64 y	219	37	43	37	68	43	108	47	
>65 y	98	17	36	31	37	24	25	11	
Sex									0.3
Total	496	84	114	19	156	27	226	38	
Male	141	24	35	25	49	35	57	40	
Female	355	60	79	22	107	30	169	48	
Race									0.7
Total	492	84	113	23	150	30	229	46	
White	386	78	88	23	120	31	178	46	
Black	40	8	12	30	12	30	16	40	
American Indian	46	9	10	22	14	30	22	48	
Other	20	4	3	15	4	20	13	65	
Ethnicity									0.5
Total	485	82	112	23	154	32	229	47	
Hispanic	17	4	1	6	7	41	9	5	
Not Hispanic	468	96	109	23	143	31	216	46	
Income									0.03 *
Total	540	92	121	22	158	29	261	48	
<$10,000	286	53	53	19	78	27	155	54	
$10,001–$15,000	117	22	33	28	40	34	44	38	
>$15,000	137	25	35	26	40	29	62	45	
Employment Status									0.5
Total	570	97	128	22	175	31	267	46	
Yes	133	23	32	24	45	34	56	42	
No	437	77	96	22	130	30	211	48	
Food Source									0.008 *
Total	588	100	133	23	177	30	278	47	
SNAP	385	65	71	18	111	29	203	53	
WIC	67	11	15	22	21	31	31	46	
Meals on Wheels	25	4	4	16	3	12	18	72	
Soup Kitchen	144	24	22	15	47	33	75	52	
HEI Score									0.02 *
	*n*	Mean ± SD	*n*	Mean ± SD	*n*	Mean ± SD	*n*	Mean ± SD	
	588	43.0 ± 12.6	133	45.4 ± 13.7	177	43.3 ± 11.9	278	41.6 ± 12.4	

Note: Totals may not add up to total participant number (*n* = 588) due to incomplete surveys. * *p*-values < 0.05 were interpreted as significantly different.

**Table 2:** Mean price metrics for total reported 24-h dietary intake among adults using rural, Midwestern food pantries recruited in 2014 ^1^.

Price Metric	Mean Price ± SD per Person (*n* = 588)			*p*-Value ^2^	Mean Price ± SD per Individual Food (*n* = 6319)			*p*-Value ^2^
	VLFS (*n* = 278) ^3^	LFS (*n* = 177) ^4^	FS (*n* = 133) ^5^		VLFS (*n* = 2707) ^3^	LFS (*n* = 2029) ^4^	FS (*n* = 1583) ^5^	
Total Price	$2.27 ± 1.64	$2.89 ± 1.94	$2.60 ± 1.66	0.004 *^,c^	$0.24 ± 0.36	$0.25 ± 0.42	$0.22 ± 0.38	0.3
Price/Gram	$0.0012 ± 0.0008	$0.0015 ± 0.0009	$0.0013 ± 0.0008	0.03 *^,c^	$0.0027 ± 0.0033	$0.0029 ± 0.0032	$0.0027 ± 0.0029	0.3
Price/Calorie	$0.0020 ± 0.0014	$0.0020 ± 0.0008	$0.0020 ± 0.0015	0.4	$0.0043 ± 0.0090	$0.0041 ± 0.0078	$0.0038 ± 0.0064	0.006 *^,b^

^1^ Derived from a single, 24-h dietary recall; ^2^ All *p*-values are after log transformation; ^3^ VLFS: Very Low Food-Secure; ^4^ LFS: Low Food-Secure; ^5^ FS: Food-Secure; ^b^ Tukey comparison: FS vs. VLFS; ^c^ Tukey comparison: LFS vs. VLFS. * *p*-values < 0.05 were interpreted as significantly different.

**Table 3:** Mean total prices for reported 24-h dietary intake by food group among adults using rural, Midwestern food pantries recruited in 2014 ^1^.

Food Group	Mean Total Price ± SD per Person (*n* = 588)			*p*-Value ^2^	Mean Total Price ± SD per Individual Food (*n* = 6319)			*p*-Value ^2^
	VLFS (*n* = 278 ) ^3^	LFS (*n* = 177) ^4^	FS (*n* = 133) ^5^		VLFS (*n* = 2707) ^3^	LFS (*n* = 2029) ^4^	FS (*n* = 1583) ^5^	
Dairy	$0.46 ± 0.42	$0.48 ± 0.47	$0.42 ± 0.42	0.1	$0.24 ± 0.34	$0.24 ± 0.25	$0.23 ± 0.20	0.4
Fruit	$0.40 ± 0.38	$0.47 ± 0.52	$0.38 ± 0.32	0.04 *^,a^	$0.29 ± 0.31	$0.31 ± 0.26	$0.26 ± 0.21	0.4
Grain	$0.50 ± 0.53	$0.58 ± 0.53	$0.53 ± 0.53	0.4	$0.23 ± 0.27	$0.24 ± 0.30	$0.21 ± 0.30	0.1
Protein	$1.00 ± 0.89	$1.21 ± 1.23	$1.17 ± 1.16	0.1	$0.56 ± 0.60	$0.62 ± 0.79	$0.57 ± 0.70	0.4
Vegetable	$0.43 ± 0.39	$0.51 ± 0.50	$0.47 ± 0.54	0.9	$0.20 ± 0.24	$0.20 ± 0.27	$0.19 ± 0.29	0.4

^1^ Derived from a single, 24-h dietary recall; ^2^ All *p*-values are after log transformation; ^3^ VLFS: Very Low Food-Secure; ^4^ LFS: Low Food-Secure; ^5^ FS: Food-Secure; ^a^ Tukey comparison: FS vs. LFS. * *p*-values < 0.05 were interpreted as significantly different.

**Table 4:** Mean price per gram for reported 24-h dietary intake by food group among adults using rural, Midwestern food pantries recruited in 2014 ^6^.

Food Group	Mean Price/Gram ± SD per Person (*n* = 588)			*p*-Value ^7^	Mean Price/Gram ± SD per Individual Food (*n* = 6319)			*p*-Value ^7^
	VLFS (*n* = 278 ) ^8^	LFS (*n* = 177) ^9^	FS (*n* = 133) ^10^		VLFS (*n* = 2707) ^8^	LFS (*n* = 2029) ^9^	FS (*n* = 1583) ^10^	
Dairy	$0.0030 ± 0.0033	$0.0031 ± 0.0023	$0.0030 ± 0.0025	0.2	$0.0034 ± 0.0033	$0.0035 ± 0.0030	$0.0032 ± 0.0028	0.4
Fruit	$0.0021 ± 0.0015	$0.0018 ± 0.0020	$0.0017 ± 0.0009	0.01 *^,c^	$0.0022 ± 0.0017	$0.0019 ± 0.0020	$0.0017 ± 0.0011	0.03 *^,c^
Grain	$0.0027 ± 0.0018	$0.0026 ± 0.0015	$0.0027 ± 0.0016	0.5	$0.0029 ± 0.0020	$0.0030 ± 0.0021	$0.0031 ± 0.0023	0.4
Protein	$0.0054 ± 0.0030	$0.0055 ± 0.0028	$0.0051 ± 0.0024	0.4	$0.0054 ± 0.0034	$0.0057 ± 0.0034	$0.0055 ± 0.0032	0.4
Vegetable	$0.0026 ± 0.0017	$0.0028 ± 0.0019	$0.0025 ± 0.0018	0.09	$0.0028 ± 0.0035	$0.0029 ± 0.0023	$0.0025 ± 0.0016	0.0001 *^,a^

^6^ Derived from a single, 24-h dietary recall; ^7^ All *p*-values are after log transformation; ^8^ VLFS: Very Low Food-Secure; ^9^ LFS: Low Food-Secure; ^10^ FS: Food-Secure; ^a^ Tukey comparison: FS vs. LFS; ^c^ Tukey comparison: LFS vs. VLFS. * *p*-values < 0.05 were interpreted as significantly different.

**Table 5:** Mean price per calorie for reported 24-h dietary intake by food group among adults using rural, Midwestern food pantries recruited in 2014 ^6^.

Food Group	Mean Price/Calorie ± SD per Person (*n* = 588)			*p*-Value ^7^	Mean Price/Calorie ± SD per Individual Food (*n* = 6319)			*p*-Value ^7^
	VLFS (*n* = 278 ) ^8^	LFS (*n* = 177) ^9^	FS (*n* = 133) ^10^		VLFS (*n* = 2707) ^8^	LFS (*n* = 2029) ^9^	FS (*n* = 1583) ^10^	
Dairy	$0.0019 ± 0.0009	$0.0019 ± 0.0008	$0.0018 ± 0.0007	0.3	$0.0019 ± 0.0010	$0.0018 ± 0.0010	$0.0018 ± 0.0007	0.8
Fruit	$0.0036 ± 0.0035	$0.0034 ± 0.0045	$0.0031 ± 0.0022	0.1	$0.0040 ± 0.0041	$0.0035 ± 0.0042	$0.0030 ± 0.0025	0.1
Grain	$0.0010 ± 0.0006	$0.0011 ± 0.0007	$0.0011 ± 0.0005	0.5	$0.0011 ± 0.0010	$0.0012 ± 0.0012	$0.0011 ± 0.0010	0.9
Protein	$0.0027 ± 0.0018	$0.0027 ± 0.0016	$0.0024 ± 0.0012	0.3	$0.0027 ± 0.0019	$0.0027 ± 0.0019	$0.0026 ± 0.0018	0.8
Vegetable	$0.0047 ± 0.0058	$0.0048 ± 0.0066	$0.0050 ± 0.0063	0.3	$0.0072 ± 0.0150	$0.0071 ± 0.0088	$0.0071 ± 0.0079	0.2

^6^ Derived from a single, 24-h dietary recall; ^7^ All *p*-values are after log transformation; ^8^ VLFS: Very Low Food-Secure; ^9^ LFS: Low Food-Secure; ^10^ FS: Food-Secure.

## Data Availability

Restrictions apply to the availability of these data. Data was obtained from the VFF team and are available with the permission of the VFF team.
